# Host Identity Matters in the Amphibian-*Batrachochytrium dendrobatidis* System: Fine-Scale Patterns of Variation in Responses to a Multi-Host Pathogen

**DOI:** 10.1371/journal.pone.0054490

**Published:** 2013-01-24

**Authors:** Stephanie Gervasi, Carmen Gondhalekar, Deanna H. Olson, Andrew R. Blaustein

**Affiliations:** 1 Department of Zoology, Oregon State University, Corvallis, Oregon, United States of America; 2 Fisheries and Wildlife Science, Oregon State University, Corvallis, Oregon, United States of America; 3 United States Forest Service, Pacific Northwest Research Station, Corvallis, Oregon, United States of America; 4 Environmental Sciences Graduate Program, Oregon State University, Corvallis, Oregon, United States of America; Smithsonian's National Zoological Park, United States of America

## Abstract

Species composition within ecological assemblages can drive disease dynamics including pathogen invasion, spread, and persistence. In multi-host pathogen systems, interspecific variation in responses to infection creates important context dependency when predicting the outcome of disease. Here, we examine the responses of three sympatric host species to a single fungal pathogen, *Batrachochytrium dendrobatidis*, which is associated with worldwide amphibian population declines and extinctions. Using an experimental approach, we show that amphibian species from three different genera display significant differences in patterns of pathgen-induced mortality as well as the magnitude and temporal dynamics of infection load. We exposed amphibians to one of four inoculation dose treatments at both larval and post- metamorphic stages and quantified infection load on day 8 and day 15 post-inoculation. Of the three species examined, only one (the Pacific treefrog; *Pseudacris regilla*) displayed “dose-dependent” responses; survival was reduced and infection load was elevated as inoculation dose was increased. We observed a reduction in survival but no differences in infection load across pathogen treatments in Cascades frogs (*Rana cascadae)*. Western toads (*Anaxyrus boreas*) displayed differences in infection load but no differences in survival across pathogen treatments. Within species, responses to the pathogen varied with life history stage, and the most heavily infected species at the larval stage was different from the most heavily infected species at the post-metamorphic stage. Temporal changes in infection load were species and life history stage-specific. We show that variation in susceptibility to this multi-host pathogen is complex when viewed at a fine-scale and may be mediated through intrinsic host traits.

## Introduction

Most pathogens can infect and be transmitted among multiple hosts, but there may be considerable variation in responses to infection among species, individuals, or between life stages [1], [2], [3], [4], [5]. Differences in host responses to pathogens can influence disease dynamics by altering the probability and rate of pathogen transmission, disease induced mortality, and recovery from infection [6], [7], [8]. Theoretical and empirical evidence suggests that variation in host competence (*i.e*. the capacity of a host to acquire, persist with, and transmit a pathogen) can modify the abundance and probability of persistence of pathogens in ecological communities [3], [9]. For example, reservoir hosts harbor and transmit a pathogen, often without incurring morbidity or mortality as a result of infection [3]. Species that act as reservoirs can facilitate long-term pathogen persistence in space and time and prevent periodic pathogen fade-outs based on density of susceptible hosts [3], [7], [9]. Therefore, species identity and composition in ecological assemblages can have a deterministic effect on pathogen invasion, spread, and persistence.

Predicting disease dynamics has become an important priority as emerging infectious diseases (EIDs) represent a major threat to global biodiversity [10], [11], [12], [13], [14] and EIDs are increasing in incidence and impact, worldwide [11], [13], [14]. Global amphibian population declines are a particularly illustrative example of the impact of EIDs on wildlife [15], [16], [17]; the fungal pathogen, *Batrachchytrium dendrobatidis* (Bd), has decimated amphibian populations, worldwide [18], [19] and is now found on every continent where amphibians exist [20], [21]. While many species die after exposure to Bd [18], other species persist and may be carriers for the pathogen [22]. On closer examination, there is likely a continuum of susceptibility to Bd among amphibian hosts [23], [24]. Where an individual or species lies along this susceptibility continuum is a product of environmental, pathogen-specific and host-specific factors, and several reviews have attempted to tease apart the most important factors driving Bd dynamics [25], [26], [27]. Intrinsic host traits may play a fundamental role in determining responses to infection and disease outcomes among amphibian populations [24], [27], [28], [29]. Species that share key life history traits, certain behaviors, and/or geographical and microhabitat preference may exhibit similar patterns of susceptibility [27], [29]. Physiological or immunological characteristics of host species may also influence the probability of infection, degree and duration of infection, infectiousness and the probability of disease-induced mortality [30], [31] and genetic traits associated with immunity may allow some individuals or groups of similar individuals to be more effective at fighting infection [32].

Bd infects keratinized structures of amphibians [33]. In larval amphibians, keratin exists in mouthparts and infection can impair feeding [34] but also may contribute to larval mortality [23], [35]. As development continues, keratin begins forming on other parts of the body; by metamorphosis animals are covered with keratinized epidermal tissue [35]. Pathogenicity of Bd results from damage to the epidermal skin layers of post-metamorphic amphibians, but also impairment of osmoregulatory mechanisms of the skin and secondary electrolyte imbalance that may lead directly to cardiac arrest in amphibians [36]. Variation in skin structure (propensity to act as an effective pathogen “barrier”) as well as variation in the amount of keratin and other proteins involved with the skin could alter host-pathogen interactions in this system. Pathogenicity may also arise from fungal metabolites, including toxic substances that cause morbidity and mortality in larval and post-metamorphic amphibians [23], [37], [38].

There is a paucity of data characterizing the individual and species-level interactions between amphibian hosts and this fungal pathogen using an experimental framework. Thus, using a comparative experimental approach, we examined responses to Bd in three sympatric host species of three genera: tree frogs (*Pseudacris regilla*), true frogs (*Rana cascadae*), and toads (*Anaxyrus boreas*). We exposed animals to one of four different inoculation dose treatments and examined mortality and infection load at both the larval and post-metamorphic stage. We sampled infection load at two distinct time-points in both larvae and metmorphs of these three species.

## Methods

### Animal Husbandry

Eggs of all species were collected with handheld dipnets from natural ponds in the Oregon Cascade Range west of Bend, Oregon (*R. cascadae* and *P. regilla*: Linn County, OR; elevation = 1140 m; *A. boreas*: Deschutes County, OR; elevation = 2300 m) during the summer of 2011 (*R. cascadae* and *P. regilla* collected in May and *A. boreas* collected July). We collected 5–10 clutches of each species to increase genetic variability in our samples, and clutches were fully mixed. Eggs were brought to a climate-controlled laboratory within 4 hours of collection and kept at 14.5–15.5°C on a natural light:dark photoperiod (regulated by an outdoor, light-sensitive receptor). After hatching, we separated tadpoles to a density of approximately 100 tadpoles per aquarium, and raised them to Gosner stage 27–30 (marked by early development of hind-limb buds [39]). During development in the laboratory, tadpoles were fed a 3:1 mixture of rabbit chow and fish food *ad libitum* (during the experiment, we fed tadpoles a pinch (approximately 0.05 g) of the same mixture of food, every other day). We changed water in larval aquaria weekly. Once the majority of larvae reached the desired Gosner stage, we randomly selected tadpoles for experiments. Larvae were housed in individual 1 L containers for the duration of the experiment and were fed one pinch of food every three days. All larvae not used in experiments were humanely euthanized by immersion in MS-222 according to approved animal care and use protocol (ACUP # 4184 Oregon State University).

For experiments with metamorphic animals, we removed free swimming stage tadpoles from the same clutches in groups of 50 to outdoor semi-natural mesocosms. These animals were kept in outdoor mesocosms for the duration of their larval and early metamorphic development. We selected tadpoles for mesocosms that were of approximately the same size and that were actively swimming in laboratory aquaria. Aquatic mesocosms were 36 inch wide×21 inch deep (∼350 L) cattle tanks, filled with well water. We added 2 L aliquots of pond water to each mesocosm. Pond water provided a natural source of algae and zooplankton to all mesocosms. We stocked each mesocosm with 100 g of dry oak leaves and 20 g of rabbit chow to provide substrate for periphyton and a source of nutrients for larvae. Mesocosms were covered with plastic screening to prevent colonization by insects and predators and were allowed to develop for 15 days before adding larval amphibians. Because of differences in breeding phenology, *A. boreas* tadpoles were raised in mescosms from July to October, while *R. cascadae* and *P. regilla* tadpoles were raised in mesocosms from May to August. As animals began to metamorphose (i.e., emergence of both forelimbs), we removed them to semi-aquatic/semi-terrestrial mesocosms containing a thin layer of water and oak leaves to prevent drowning. Animals were maintained at a density of approximately 100 animals per mesocosm and were not maintained separately by larval/aquatic mesocosm (i.e., individuals within the same species were mixed as they entered semi-terrestrial mesocosms). Animals in terrestrial mesocosms were allowed to complete metamorphosis (complete tail absorption) and remained in these enclosures for 2–3 months after metamorphosis. After metamorphosis, animals were fed pinhead crickets and wingless fruit flies weekly, *ad libitum*. After 2–3 months of development, metamorphic amphibians were brought into the same laboratory where larval experiments were run. Animals were allowed to acclimate for 48 hours before they were weighed, measured, and assigned to their experimental treatment. All metamorphic animals from terrestrial mesocosms were re-randomized for subsequent experiments in the laboratory. Metamorphic amphibians were housed in individual large-sized (140×30 mm) petri dishes throughout the laboratory experiment and were fed pinhead crickets by average body weight (1 cricket per 0.1 g body mass; same number of crickets for each animal) twice a week.

### Inoculate Preparation

*Batrachochytrium dendrobatidis* isolate, JEL 274 originally isolated from *A. boreas* from Colorado [40], was cultured on 100 mm×15 mm tryptone agar plates and allowed to grow for 6 days at 23°C before inoculation of animals. Previous studies have shown that zoospore activity and density with this particular strain of Bd are highest within 6–8 days after culturing the pathogen on tryptone agar plates [24]. Zoospores were harvested by flooding agar plates with 10 ml of dechlorinated water and scraping the surface of the agar before pooling the inoculums of several (5–6) plates [24]. Experimental animals were exposed to 10 ml of pooled inoculation broth at a density of 100,000 zoospores total (“high” inoculation dose), 50,000 zoospores total (“intermediate” inoculation dose), or 10,000 zoospores total (“low” inoculation dose). All zoospore counts were determined by hemocytometer from pooled inoculation broth. For larval amphibians, zoospores were transferred into individual plastic cups, already containing 600 ml of dechlorinated water. In metamorphic amphibians, zoospores were transferred to individual large petri dishes, already containing 15 ml of dechlorinated water. Control animals were exposed to the same volume of sham inoculation (created from pathogen-free tryptone-agar plates). Thus, throughout the experiment, a thin layer of inoculated water was present at the bottom of the petri dish. Animals resting on the bottom of petri dishes were exposed to but not immersed in the diluted inoculate and could climb the walls and tops of Petri dishes. This method standardized pathogen exposure regimes among amphibian species, allowing us to assess baseline differences in species responses to the same treatment regime.

### Experimental Pathogen Exposure

For both larval and post-metamorphic animals, the experimental duration was 15 days. Animals were exposed to their treatments once, at the start of the experiment and then observed daily. We did not to re-inoculate animals over the course of the infection because we were specifically testing how infection load changes over the time-frame of a single exposure event. We assessed infection loads in larval and post-metamorphic animals on day 8 and day 15. We only sampled live animals at each time-point, since pathogen growth, and thus, infection load could change rapidly on animals that have died and begin to immediately decompose. Thus, we established how infection load differs on day 8 versus day 15 in living animals, exposed once to a standardized inoculation dose of Bd.

### Larval Pathogen Exposure

We staged all larvae immediately prior to initiation of the experiment, and then placed Gosner stage 27–30 animals [39] in individual 1 L plastic cups (the experimental unit), containing 600 ml of water. Larvae were randomly assigned to one of four treatment groups (n = 30): High Bd dose, Intermediate Bd dose, Low Bd dose, or Control (no Bd). We checked for mortality daily, and preserved dead animals in 95% ethanol. On day 5 of the experiment, we increased the water level to 900 ml to increase oxygen content in the cups. On day 8 of the experiment, we randomly sampled 15 individual tadpoles in each experimental treatment for quantitative PCR and changed all remaining (non-sampled) animals into clean water. At the end of the experiment on day 15, the remaining animals were humanely euthanized in accordance with the institutional animal care protocol and preserved in 95% ethanol. Post-experimental snout-vent-length and mass were taken only for tadpoles that were sampled on day 8 and day 15 for quantitative PCR.

### Post-metamorphic Pathogen Exposure

All animals from semi-terrestrial outdoor mesocosms went through 48 h of acclimation in individual Petri dishes to laboratory conditions before initiation of the experiment. At the start of the experiment, all animals were weighed, measured, and randomly assigned to one of four treatment groups, identical to treatments for larvae. For metamorphic *R. cascadae* and *P. regilla*, 30 animals were assigned to each treatment. For metamorphic *A. boreas*, 20 animals were assigned to each treatment, except in the high dose treatment (n = 21). As with larval amphibians, we checked for mortality daily. On day 8 of the experiment, we sampled half of all remaining animals for qPCR and changed the water in each petri dish (without re-inoculating animals). At the end of the experiment on day 15, the remaining animals were humanely euthanized in accordance with institutional animal care protocol and preserved in 95% ethanol.

### Quantification of Infection Load

For both larval and post-metamorphic amphibians, we used quantitative-PCR (qPCR) to assess infection load in larval and post-metamorphic amphibians following the methods of Boyle et al. [41], except that we used 60 µl of Prepman Ultra (Applied Biosystems) instead of 40 ml in all DNA extractions. Extractions were diluted 1:10 and processed in an ABI PRISM 7500 (Applied Biosystems). Whole mouthparts were extracted for qPCR in larvae. For metamorphic amphibians, ventral abdominal skin and inner thigh skin was swabbed using fine tipped sterile rayon swabs (Medical Wire and Equipment- MW&E 113) for Bd. We sampled 15 larval amphibians for qPCR in each pathogen-exposed treatment on day 8 of the experiment and 8 larval animals from each pathogen-exposed treatment on day 15 of the experiment. In metamorphic amphibians we sampled half of all surviving animals for qPCR on day 8 of the experiment and the remaining animals were sampled on day 15. Each sample was analyzed in triplicate and the average number of genome equivalents of Bd per animal was calculated. For larval and post-metamorphic amphibians, we also randomly sampled at least 3 control animals at each time-point and in each treatment. All control animals (larval and post-metamorphic) tested negative for infection in all three replicate DNA wells for quantitative PCR.

### Statistical Analyses

Statistical comparisons were made among treatments (for each species, individually), among species (at each treatment level, separately), and between time-points (day 8 versus day 15). Each of level of statistical comparison (treatment, species, time-point) was carried out individually, as opposed to a single analysis with multi-level comparisons. We used Kaplan-Meier (or product-limit) analyses in S-plus version 8.0 for Windows to generate “survival curves” among species and among treatments. This non-parametric method allows the analysis of survival data that is often right skewed. In addition, Kaplan-Meier analyses allow for “censored data”, which accounts for sub-sampled individuals in our experiment that were “lost” or “removed” due to destructive sampling (not to death). This was important in our dataset, since a subset of live animals were sampled (i.e., removed from the experiment) on day 8 of the experiment for a quantitative assessment of infection load. Thus, the survival curves generated take into account mortality as a constant function of the remaining (but not sub-sampled) individuals. To statistically compare survival curves, we used a Cox’s proportional hazards model [42]. The Cox proportional hazards model give an overall p-value (Likelihood ratio test) which assesses the validity of the model, as well as p-values for each factor and an associated “hazard ratio”. The hazard ratio represents a comparative indicator of the risk or probability of mortality associated with a given factor (a hazard ratio>1 indicates an increase in the probability of mortality). Higher hazard ratios are associated with a greater probability or risk of mortality due to association with that factor (e.g., treatment or species). One caveat with Cox proportional hazards models for survival analysis is that some mortality (greater than 1 individual) must exist to make comparisons among groups. Thus, if zero mortality is seen in a group, a comparison between that group and others is not possible. We only experienced this problem in tadpoles where, for one species, no mortality existed across all experimental treatments, and in one treatment for a single post-metamorphic species (i.e., zero mortality in the control treatment for *A. boreas* metamorphs). For all analyses with post-metamorphic amphibians, our proportional hazards models also included initial body mass as a factor. Survival analyses of larval amphibians were performed only with main effects (treatment or species), as there was no *initial* metric of body size or mass available; we only had post-experimental estimates of size and mass on the subset of animals sampled for quantitative-PCR analysis). Cox proportional hazards models were performed in R, statistical computing environment (version 2.9.0, Institute for Statistics and Mathematics, Vienna) with the “coxph” function and the Survival package for survival analyses within and among species.

For infection load analyses, we transformed quantitative-PCR loads (log-average genome equivalents per individual +1) to normalize data and used a one-way analysis of co-variance to examine within species (among-treatment) and among species (within-treatment) differences in infection load in larval and post-metamorphic animals. We used treatment or species as the main effect and included mass as a covariate in all models (since infection load may depend on the amount of host-body-area or mouthpart areas that the pathogen can colonize). We used a Levene’s test to check for homogeneity of variance among groups (treatments or species) before running all ANCOVA’s (and in all cases, we met the requirements of the parametric test). When we detected a significant overall effect of treatment or species, we carried out individual pair-wise comparisons to control type I error using a Tukey’s honestly significant difference test. In the case of low sample size (for larval or post-metamorphic amphibians some species/treatment combinations had only one individual because of lack of Bd-positive animals or because of high mortality during the experiment) we used Welch’s modified t-tests to make between group comparisons. This t-test controls for non-equal variance between groups. We also examined temporal dynamics of infection by comparing quantitative-PCR loads on day 8 of sampling versus day 15 of sampling within treatments and across species using Welch’s modified t-tests.

### Ethics Statement

This study was carried out in strict accordance with the recommendations in the Guide for the Care and Use of Laboratory Animals of the National Institutes of Health. Animals in the study were allowed to die directly as a result of infection and this is in accordance with the approved Institutional Animal Care and Use Committee of Oregon State University (ACUP # 4184). Criteria for euthanasia included display of overt signs of morbidity associated with infection (loss of righting reflex and anorexia in post-metamorphic animals and extreme weakness in larval amphibians). In our study, we checked animals twice, daily, for these signs of morbidity and were humanely euthanized if we observed signs (although we did not observe these extreme signs of morbidity in our two week experiment). Any animals appearing to show any signs of distress were immediately euthanized in MS-222 according to institutional animal care protocol. Collection of amphibian eggs for all experiments was approved by the Oregon Department of Fish and Wildlife (2011 Oregon Scientific Taking Permit #006-12 issued to A.R. Blaustein).

## Results

### Larval Survival

There were no differences in rates of mortality across experimental treatments, including the control treatment, for any larval species (Table 1). We also failed to detect species-level differences in survival (*R. cascadae* versus *P. regilla* only) within pathogen treatments (Table 1). We did not include *A. boreas* in the among species comparisons because we observed zero mortality across all treatments, including control and pathogen exposures, for larval *A. boreas* and this precludes the ability to use a Cox proportional hazards model.

**Table 1:** Treatment and species level effects of *Batrachochytrium dendrobatidis* exposure on larval and post-metamorphic stage survival of three amphibian species (Cox Proportional Hazards models).

A. Larval Stage	Level	Overall Effect	Comparison	Hazard Ratio	p-value
	Treatment	LRT = 7.4; 3df;p = 0.06	PR	NA	All >0.05
		LRT = 1.79; 3df; p = 0.62	RC	NA	All >0.05
		NA*	AB	NA	NA
	Species	LRT = 2.13;1df;p = 0.144**	H	NA	All >0.05
		LRT = 0.08;1df;p = 0.770**	I	NA	All >0.05
		LRT = 1.1; 1df;p = 0.294**	L	NA	All >0.05

B. Post-metamorphic Stage	Level	Overall Effect	Comparison	Hazard Ratio	p-value
	Treatment	LRT = 76.3;4df;p<0.0001	PR – L vs C	1.77	0.396
			PR- I vs C	11.1	<0.0001
			PR – H vs C	32.0	<0.0001
			PR – I vs L	6.30	0.0002
			PR – H vs L	18.0	<0.0001
			PR – H vs I	2.87	0.0004
		LRT = 40.9;4df;p<0.0001	RC – L vs C	0.602	0.720
			RC- I vs C	4.0	0.199
			RC – H vs C	8.5	0.041
			RC – I vs L	6.65	0.080
			RC – H vs L	14.1	0.011
			RC – H vs I	2.12	0.145
		LRT = 4.96;3df;p = 0.175	NA	NA	All >0.05
	Species	LRT = 29.3;3df;p<0.0001	H - PR vs RC	3.08	0.006
			H - PR vs AB	2.64	0.007
			H - AB vs RC	1.17	0.734
		LRT = 24;3df;p<0.0001	I - PR vs RC	3.12	0.020
			I - PR vs AB	3.75	0.003
			I - RC vs AB	1.20	0.768
		LRT = 12.2;3df;p = 0.006	L - PR vs RC	4.3	0.196
			L - AB vs PR	2.8	0.07
			L - AB vs RC	12.3	0.02

Abbreviations are used to denote species identify and treatments: PR = *Pseudacris regilla*; RC = *Rana cascadae*; AB = *Anaxyrus boreas*; H = high dose; I = intermediate dose; L = low dose. NA indicates that the comparison is not applicable because it is precluded by a non-significant overall effect in the model. For all hazard ratios, the comparison is the more extreme group (treatment or species) compared to the less extreme group (i.e., the risk of being in the more “severe”, or higher infectious dose treatment is given and the more “severe” treatment is listed first). LRT = Likelihood ratio test.

* Indicates no statistical comparison of survival rate across larval *Anaxyrus boreas* treatments due to zero mortality in all treatments (control, low, intermediate, and high).

** Indicates comparisons made between *Rana cascadae* and *Pseudacris regilla* because there was zero mortality in *Anaxyrus boreas* precluded inclusion in Cox proportional hazards model.

### Larval Infection Load

#### Pseudacris regilla

**Figure 1:**
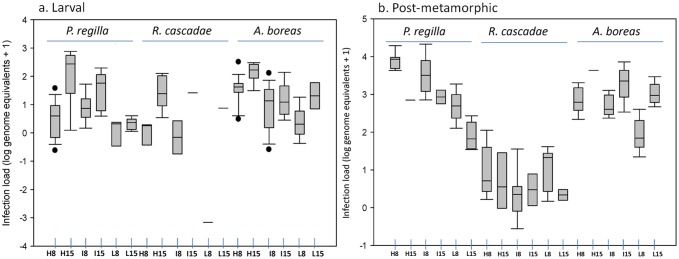
Box plots showing pathogen infection load (log genome equivalents +1) of larval and post-metamorphic amphibians across species, treatments, and sampling time-points. We examined infection load of larval (a.) and post-metamorphic (b.) amphibians of three species (*Pseudacris regilla, Rana cascadae*, and *Anaxyrus boreas*) exposed to a High (H), Intermediate (I), or Low (L), inoculation dose treatment and sampled either on day 8 or day 15 of the experiment. Only live animals were sampled at each time-point. Low sample sizes (1 individual) are indicated with a bar and represent a low number of Bd-positive animals or low sample size due to mortality. Lines in the boxes represent the “mean” infection load. Bars are 2 SE of the mean (+/−) and outliers are represented by black dots.

Although we did not detect an effect of the pathogen on survival, we did observe significant differences in infection load in larval amphibians dependent on treatment, species, and/or sampling time-point (Table 2; Table S1; Fig. 1.a.). For *P. regilla* tadpoles there were significant differences in infection load among experimental pathogen inoculation treatments, but only at the day 15 time-point (ANCOVA F_3,14_ = 5.93;p = 0.013); individuals in the high pathogen inoculation treatment had significantly greater infection loads than individuals in the low pathogen inoculation treatment (Tukey HSD test p<0.05). Infection loads were different on day 8 versus day 15 in *P. regilla*, but only at the high inoculation dose treatment; infection load was higher on day 15 than day 8 of the experiment (Welch’s 2-sample t-test; t_15_ = −3.147; p = 0.011). Although infection load increased over time in *P. regilla* tadpoles in the high inoculation dose treatment, the percentage of animals testing positive for infection at this high treatment level went from 91% on day 8 to 87% on day 15. Conversely, the percentage of animals testing positive for infection increased in the two other inoculation dose treatments, from 67% to 75% in the intermediate dose treatment and from 28% to 62% in the low dose treatment from day 8 to day 15.

**Table 2:** *Batrachochytrium dendrobatidis* infection load summary for larval and post-metamorphic anurans of three species.

Species	Day	Treatment	Average genome equivalents +/− SD Larval	Average genome equivalents +/− SD Post-metamorphic
PR	8	High	0.798 (+/−1.13)	927(+/−657)
		Intermediate	1.77(+/−2.79)	691(+/−938)
		Low	0.170 (+/−0.112)	73.2 (+/−71.1)
	15	High	32.4 (+/−30.1)	71.0*
		Intermediate	7.80 (+/−7.99)	93.2 (+/−52.1)
		Low	0.234(+/−0.117)	11.2 (+/−10.0)
RC	8	High	0.134 (+/−0.097)	3.12 (+/−4.98)
		Intermediate	0.144 (+/−0.177)	1.02 (+/−2.22)
		Low	0.00007*	1.96 (+/−1.59)
	15	High	5.30(+/−5.55)	2.13 (+/−3.41)
		Intermediate	2.62*	0.448(+/−0.470)
		Low	0.750*	0.229 (+/− 0.101)
AB	8	High	6.75(+/−10.5)	91.3 (+/−75.9)
		Intermediate	2.76 (+/−3.94)	60.9 (+/−43.8)
		Low	0.579 (+/−0.838)	13.9 (+/−16.0)
	15	High	17.6(+/−10.6)	433*
		Intermediate	4.16 (+/−6.29)	295 (+/−271)
		Low	3.34 (+/−3.73)	132 (+/−99.8)

Average genome equivalents (un-transformed) given for comparison among species, treatments, and across sampling time-points (day 8 and day 15).

* No standard deviation given for these measures because there was only 1 individual remaining or 1 individual testing positive for infection in this treatment/species combination.

#### Rana cascadae

Larval *R. cascadae* showed no differences in infection load between experimental treatments on day 8, and only one individual in the low treatment group tested positive for infection (Fig. 1.a.). On day 15, a single intermediate treatment animal and a single low treatment animal tested positive for infection; thus, we could not make statistical comparisons among treatments on day 15 for larval *R. cascadae*. Examination of the high inoculation treatment, where sample size allowed statistical comparisons between time-points, showed a change in infection load from day 8 to day 15 of the experiment; infection load increased between the sampling time-points (Welch’s 2 sample t-test; t_5_ = −3.423; p = 0.018). The percentage of animals sampled that tested positive for infection from day 8 to day 15 went from 21% to 55% in the high inoculation treatment.

#### Anaxyrus boreas

In larval *A. boreas*, infection load was significantly different among pathogen inoculation treatments on day 8 of the experiment (Fig. 1.a.; ANCOVA F_3,27_ = 6.47;p = 0.005). A Tukey’s HSD test revealed that infection loads were significantly greater in high inoculation treatment animals than low inoculation treatment animals (p<0.05). Sampling of larval toads on day 15 also revealed a significant difference in infection load among treatments (ANCOVA F_3,12_ = 5.18;p = 0.023); animals in the high pathogen inoculation treatment had higher levels of infection than animals in the intermediate inoculation treatment (p<0.05). Infection load in larval toads was higher on day 15 compared to day 8, but only significantly so in the high inoculation treatment (Welch’s 2-sample t-test: t_18_ = −2.82; p = 0.012). For *A. boreas* tadpoles, the proportion of animals testing positive for infection in the high inoculation treatment stayed at 87% from day 8 to day 15; in the intermediate inoculation dose treatment went from73% on day 8 to 87% on day 15; and went from 46% on day 8 to 25% on day 15 in the low inoculation dose treatment.

#### Among-Species

We detected a significant effect of larval species identify on infection load, but only on day 8 of the experiment and at the high inoculation dose treatment (Table 2; Table S1; ANCOVA F_3,22_ = 12.7; p = 0.0002); infection loads were significantly higher in *A. boreas* than *R. cascadae* (p<0.05) and significantly higher in *A. boreas* than *P. regilla* (p<0.05). Overall, on day 8, *A. boreas* had the highest infection loads and *R. cascadae* had the lowest infection loads.

### Post-Metamorphic amphibians

#### Pseudacris regilla

**Figure 2:**
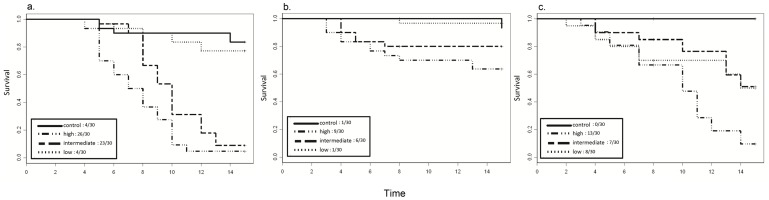
Kaplan-meier survival curves for among treatment responses of post-metamorphic animals to *Batrachochytrium dendrobatidis* treatments. For *P. regilla* (a.) the rate of mortality was significantly different among all three pathogen treatments (low, intermediate, and high inoculation dose). There were no significant differences between rates of mortality in the control versus the low inoculation dose treatment. For *R. cascadae* (b.) the rate of mortality was significantly greater in the high inoculation dose treatment compared to the low inoculation dose and control treatments. All other between treatment comparisons were non-significant. For *A. boreas* (c.) there were no significant differences in rate of mortality among the three pathogen treatments. Absolute mortality out the total number animals in each treatment is given in the in the figure legend boxes.

*P. regilla* had reduced survival as treatment level (*i.e*., inoculation dose) increased (Fig. 2.a., Table 1; Cox Proportional Hazards Model likelihood ratio test  = 76.3 on 4 df; p<0.0001). Survival was significantly different among all pathogen treatment levels (Table 1; intermediate versus low p = 0.0002; high versus intermediate p = 0.0004; high versus low p<0.0001). Survival in the low inoculation treatment was not significantly different from survival in the control treatment (p = 0.396), but survival in the intermediate and high treatments was reduced compared to survival in the control treatment (p<0.0001 and p<0.0001 for the intermediate versus control and high versus control treatments, respectively). The effect of mass on survival was also significant in the Cox model (p<0.0001). Day 8 infection load on live (i.e. sub-sampled) *P. regilla* varied with inoculation dose treatment (Fig. 1.b.; Table 2; Table S2; ANCOVA; F_3,19_ = 14.4;p = 0.0001); infection load increased with increasing inoculation treatment between the high and low (p<0.05) and intermediate and low treatments (p<0.05). Mass was not a significant factor in the infection load model (p = 0.78). By the second sampling time-point on day 15, only 1 post-metamorphic individual remained alive in the high inoculation dose treatment, so we compared infection load between the intermediate and low treatments only. Infection load was higher in the intermediate inoculation dose treatment than the low treatment (Welch’s t-test t_6_ = 4.314; p = 0.029). Infection load in sub-sampled *P. regilla* decreased within all treatments between day 8 and 15; this negative change in infection load was significant in the intermediate treatment (Welch’s t-test t_8_ = 3.568; p = 0.004) and was marginally significant in the low inoculation dose treatment (Welch’s t-test t_12_ = 2.22; p = 0.09). It was not possible to make a statistical comparison for the high treatment since only 1 individual was present on day 15. All post-metamorphic *P. regilla* individuals sampled for infection tested positive for infection on both day 8 and day 15.

#### Rana cascadae

*Rana cascadae* displayed reduced survival across treatment levels (Fig. 2.b.; Table 1; Cox Proportional Hazards Model likelihood ratio test  = 40.9; 4df; p<0.0001), but the only significant difference existed between individuals in the high versus low treatment group (p = 0.011) and high versus control treatment (p = 0.041). Mass was also a significant predictor of survival in this model (p<0.0001). Although inoculation treatments differed by an order of magnitude (100,000 versus 10,000 zoospores), infection load in *R. cascadae* metamorphs did not differ among inoculation dose treatments on day 8 or day 15 (Figure 1.b.; Table 2; Table S2). Further, there was no significant difference in infection load on day 8 versus day 15 in the high and intermediate treatments, although there was a significant decrease in infection over time in the low inoculation dose treatment (Figure 1.b.; Welch’s t-test t_5_ = 3.568;p = 0.018). *Rana cascadae* was the only post-metamorphic species to show less than 100% infection-positive sampled animals on day 8 and day 15. In *R. cascadae* on day 8, 100% of the high inoculation dose treatment animals sampled for infection were positive, while 87% of intermediate treatment animals were positive and 85% of low treatment animals were positive. On day 15 these percentages dropped; 67% of high dose treatment animals tested positive for infection, whereas only 25% of animals tested were positive for infection in both the intermediate and low treatment groups on day 15.

#### Anaxyrus boreas

Survival in *A. boreas* did not vary across pathogen inoculation treatments (Fig.2.c., Table 1) and mass was not a significant factor in this model. In the within species analysis, we did not include a comparison of survival between the control and pathogen exposed treatments because there was zero mortality in the control treatment for this species, precluding this treatment from the Cox model. Although there were no survival differences among the three pathogen treatments, there was a significant effect of treatment on infection load in *A. boreas* on day 8 (Fig. 1.b; Table 2; Table S2; ANCOVA; F_3,17_ = 14.5; p = 0.0002); infection load in the high inoculation dose treatment was greater than infection load in the low inoculation dose treatment (p<0.05) and infection load in the intermediate treatment was greater than infection load in the low treatment (p<0.05). There were no significant differences in infection load among treatments on day 15. Further, infection load was greater on day 15 than day 8 at all three treatment levels (Fig. 1.b.; Table 2; Table S2). While a statistical comparison of day 8 versus day 15 infection load was not possible in the high treatment (only one individual remained on day 15), infection load was significantly greater in the intermediate (Welch’s t-test t_11_ = −2.48; p = 0.038) and low inoculation treatment (Welch’s t-test t_10_ = −4.868; p = 0.0007) on day 15, compared to day 8. All post-metamorphic *A. boreas* sampled for infection tested positive for the Bd at both time-points.

#### Among-Species Comparisons

We observed significant differences in mortality rates between *P. regilla* and *R. cascadae* and between *P. regilla* and *A. boreas* at all treatment levels (Table 1). In the high (Cox Proportional Hazards Ratio likelihood ratio test  = 29.35 on 3df, p<0.0001) and intermediate (likelihood ratio test  = 24 on 3df, p<0.0001) inoculation dose treatments, all pair-wise comparisons between species p-values were <0.05; see Table 1 for p-values among species at each treatment level). At the low inoculation dose level, there were among species differences in survival (likelihood ratio test  = 12.2 on 3df, p = 0.006), but the only significant pair-wise difference was between *R. cascadae* and *A. boreas*. Mass was a significant factor in the Cox model for the high (p = 0.0007) and intermediate (p = 0.012) treatment level, but not at the low treatment level (p = 0.521). Infection load also varied among species on day 8; each species exhibited significantly different infection loads at the high inoculation dose treatment (Fig. 1.b.; Table 2; Table S2; ANCOVA F_3,18_ = 61.1; p<0.0001; all pair-wise comparisons p<0.05), the intermediate inoculation dose treatment (ANCOVA F_3,18_ = 58.5; p<0.0001), and the low inoculation dose treatment (ANCOVA F_3,17_ = 17.2;p<0.0001). On day 8, the highest infection loads were observed in *P. regilla* and the lowest infection loads were observed in *R. cascadae*. There was almost a 100 fold difference in average infection load between these two species (Table 2; average infection load in the high treatment for *P. regilla* = 927 genome equivalents (SD+/−657) versus average infection load in the high treatment for *R. cascadae* = 3.12 genome equivalents (SD+/−4.98) (Table 2). Because of low sample sizes, we could not make among species comparisons at the high treatment level on day 15. However, there were significant differences in infection load among species at the intermediate (ANCOVA F_3,6_ = 28.3; p = 0.0008) treatment level (p<0.05 for *A. boreas* verus *R. cascadae* and *P. regilla* versus *R. cascadae*) and low treatment levels (ANCOVA F_3,9_ = 59.3; p<0.0001; p<0.05 for *A. boreas* versus *P. regilla* and *A. boreas* versus *R. cascadae*). Overall, *A. boreas* showed the highest infection loads on day 15, followed by *P. regillla*. Again, on day 15, infection loads were lowest in *R. cascadae*.

### Larval versus Metamorphic Stage Comparisons

Although we did not examine the relationship statistically, as expected, there was a qualitative difference in the effect of the pathogen on survival between amphibian life history stages (Table 1); larval amphibians of all species had greater survival than all post-metamorphic animals. There was no evidence for any significant pathogen effect on survival in larval amphibians. Temporal dynamics of infection varied between stages as well. While we detected greater infection loads in larvae on day 15 than day 8, changes in infection load from day 8 to day 15 were different in each post-metamorphic species we examined, dependent on species affiliation,. In general, infection loads were lower in larval species than in post-metamorphic amphibians (Table 2) but there were exceptions. For example, infection loads in larval *A. boreas* and *P. regilla*, in particular by day 15 of the experiment, were greater than infection loads in post-metamorphic *R. cascadae* (Table 2). Qualitatively, we note that infection loads in larval and post-metamorphic *R. cascadae* were similar. Thus, for at least one species, the zoospore load localized in tadpole mouthparts was roughly equal to the amount of infectious particles found on the surface of the skin in post-metamorphic animals (Table 2).

## Discussion

Three co-occurring amphibian species exhibited very different responses during experimental exposure to the fungal pathogen, *Batrachochytrium dendrobatidis*. Variation in responses was pronounced at the species level, treatment level, between life history stages, and between sampling time-points. We show that intrinsic host traits, alone, can be important mediators of pathogen dynamics in the amphibian-chytrid fungus system. Similarly, temporal and dose-dependent responses of hosts could affect species persistence in natural environments and alter species composition in ecological assemblages.

Previous studies have shown that some species are very susceptible to Bd and succumb to mortality soon after exposure, [24], [43] while other species show no signs of morbidity or mortality in the presence of the pathogen [22]. However, increasing evidence, including results from this study, show that many species fall within the middle of this susceptibility continuum, with pronounced variation in morbidity, mortality, and infection load [24], [44]. This study shows that the host pathogen interaction is complex in the amphibian-Bd system. Among the three species examined in this study, survival and infection load were not always correlated with the magnitude of our pathogen exposure regimes (i.e., level of zoospore dosage at the low, intermediate, or high level). In one species, although the rate of mortality increased with the magnitude of pathogen exposure, the host infection load did not change with increased pathogen exposure. Conversely, a treatment-level increase of infection load in larval and post-metamorphic hosts was not always associated with greater mortality over time. In our study, larval infection load differed among species and/or treatments, yet an increase in infection did not translate into increased mortality in the larvae of any species. This is in contrast to previous studies that have shown mortality at the larval stage for some species (e.g., [23], [35]), although other studies have shown low, or no mortality at the larval stage [45]). Direct comparisons to these studies are difficult because of differences in methodology. Thus, at both the larval and post-metamorphic stage, individual host responses play a central role in determining the outcome of this host-pathogen interaction.

The possibility of a “threshold” zoospore load, above which mortality occurs [46] was only evident in one species, *P. regilla*. In this species, average infection load in the high treatment group was much higher than for any other species/treatment combination; it was greater than an order of magnitude higher in this species than the “least infected” species (*R. cascadae*). These results suggest that post-metamorphic *P. regilla* may be an important source of or reservoir for Bd in their environments [47]. For example, we did not see a difference in mortality above background control mortality levels in the low inoculation dose treatment for *P. regilla*, but infection loads at this level of exposure were high compared to low dose infection loads for the other species examined (Table 2, day 8 average genome equivalents for *P. regilla* = 73.22 SD = 71.13). Thus, *P. regilla* may be tolerant of low level infections, but not necessarily resistant to the pathogen (i.e., not effective at preventing pathogen replication in their skin). *P. regilla* has been cited as a potential “reservoir” species for Bd in other geographic contexts [47] and our experimental results support this hypothesis. This species is a ubiquitous generalist species in coastal, valley, and montane sites across Western North America, where many amphibian assemblages may be exposed to Bd via this species’ “reservoir”. Consequently, *P. regilla* may have a severe impact on the spread and maintenance of infection across a broad geographic area.

Responses to infection in the other two species that we examined were not dose dependent. For these species, there may be a much lower infection load above which mortality occurs. Or, there may be environmental or physiological mechanisms that prevent this threshold of infection from being reached. In *R. cascadae*, the rate of mortality increased with the magnitude of pathogen exposure (i.e., treatment), however, absolute mortality in *R. cascadae* was low compared to the two other species examined (Fig. 2). Although there was a significant effect of the pathogen on rate of survival in *R. cascadae*, infection load was not different among treatments at either sampling time-point for this species. *Rana cascadae* always showed the lowest infection loads (at both larval and post-metamorphic stages), the lowest percentage of Bd-positive animals, and the least amount of change in infection load over time. The reduced overall mortality and low infection loads suggest possible tolerance and resistance mechanisms may be at play in post-metamorphic *R. cascadae*. That is, individuals may be resisting infection, minimizing pathogen replication, or tolerating infection by minimizing the damage caused by the pathogen (i.e., mortality) across treatments [47], [48], [49]. Infection loads in larval *R. cascadae* on day 15 were comparable to infection loads of post-metamorphic animals of the same species. We did not observe this trend in the two other species, likely because infection is generally much higher when the amount of area the pathogen can colonize (the metamorphic skin versus larval mouthparts) was greater. The results for *R. cascadae* contrast to what we observed in *A. boreas*. For this species, the rate of mortality was the same regardless of pathogen treatment; even though animals were exposed to 10,000 versus 100,000 zoospores in the low versus the high pathogen treatment group, mortality over the course of the experiment was the same. That is, if *A. boreas* was exposed to the pathogen, they appeared to suffer pathogen-induced mortality, regardless of infectious dose (i.e., they were equally “intolerant” of the pathogen [48], [49]). However, this same species showed significant differences in infection load accrued on the skin; infection loads were greater in the higher pathogen inoculation treatments. We note that survival trends in *A. boreas*, more than either of the other two species, appeared to be temporally variable; mortality among pathogen treatments pre-day 10 of the 15 day experiment was overlapping and similar (Figure 2.c.). However, post-day 10, there was a clear separation of mortality trends between the more and less severe (high and low) inoculation treatments. This trend may suggest latency to respond in this species, and therefore, potential for the pathogen to get a foot-hold before the host has a chance to respond or combat infection.

Of importance is the temporal response to infection in larval and post-metamorphic frogs. In larvae of all species tested, infection loads in the keratinized mouthparts of tadpoles were greater on day 15 versus day 8. However, temporal responses to infection varied among each species post-metamorphosis. For post-metamorphic *P. regilla*, infection load decreased over time. In *A. boreas*, infection load increased from day 8 to day 15. In *R. cascadae*, infection load stayed the same over time (with the exception of individuals in the low treatment group where infection decreased over time). These three different temporal responses have implications for host-maintenance of the pathogen in their environment but also imply possible differences in the mechanisms by which hosts respond to and deal with infection. A decrease in infection load over time may simply be a consequence of animals with the highest infection load being removed from sampling through death (with the least heavily infected animals sampled on day 15). However, it is also possible that behavioral or physiological defenses provided a way to respond to or avoid the pathogen. Although not specifically quantified, we observed that *P. regilla* spent most of the time out of direct contact with water and zoospores, instead, climbing the sides and tops of their experimental containers. This behavioral response may have limited their contact with infectious zoospores, and decreased infection load on post metamorphic animals over time. One study suggests that the skin architecture of *P. regilla* may allow it to spatially separate areas of infection across the skin, and minimize the damage caused by infection [47]. The increasing infection load observed in post-metamorphic *A. boreas* could be a result of the lack of physiological or immunological mechanisms that impair or reduce infection load. Overall low infection loads were observed in both larval and post-metamorphic *R. cascadae*. Perhaps this species keeps infection “in check” with active cellular or humoral responses that effectively kill the pathogen or render it unable to replicate.

As shown in previous studies [50], *A. boreas*, harbored the highest infection load in larval mouthparts after initial pathogen inoculation (although, interestingly, this was the smallest species in size as larvae and thus, had a smaller area for the pathogen to infect). This suggests stage specific or life history variation in the potential for species to serve as pathogen reservoirs; amphibians have different effects on pathogen dynamics, dependent on species and stage. In *P. regilla*, as in all species examined at the larval stages, infection load increased over time. This was surprising since no re-inoculation took place between the first and second sampling time-points. In anuran larvae, infection is restricted to the keratinized mouthparts (toothrows) and can cause sublethal effects, including reduced foraging capacity [34]. We did not observe significant mortality among pathogen treatments at the larval stage in this or any of the other amphibian species, and so, we hypothesize that a mortality effect may be delayed until infection load reaches higher levels in the mouthparts, and begins to interfere with growth and or development. Infection load may accrue, uninhibited in tadpoles; perhaps the localized nature of infection precludes immunological responses to diminish infection load and infection therefore increases unabated.

Scaling from the individual to the ecological level, we emphasize the need to understand the relative contribution of species-level responses in shaping the composition and persistence of ecological assemblages. In addition to the ability of hosts to accumulate infection and persist over time with infection, there are important life history traits (lifespan, aggregation behavior, breeding phenology, migratory patterns, aquatic tendency, etc.) and density dependent factors that could interact to affect how the pathogen is spread and maintained over space and time in any given system [46], [51], [52]. For example, schooling of larval *A. boreas* in groups of thousands of individuals and synchronous metamorphosis in both *R. cascadae* and *A. boreas* results in high densities of individuals that may facilitate transmission of pathogens [53]. Furthermore, environmental factors may reduce or amplify the relative contribution of each host to pathogen systems. For example, temperature may interact with host responses to infection [54]; immunological changes occurring at warmer or cooler temperatures could promote or reduce pathogen replication in mouthparts of skin of larval or post-metamorphic amphibians or change patterns of host resistance against infection [54]. Similarly, temperatures that promote host shedding of the pathogen may speed up recovery time or allow persistence of the host with continuous low level infection. We emphasize the need for “ground-truthing” the laboratory results found here. Specifically, we encourage a quantitative assessment of infection loads in individual species existing in different combinations with other species in ecological assemblages across landscapes. Macro- and microhabitat variation represent important sources of covariates in such analyses and important correlations may exist between biotic and abiotic factors and disease dynamics in the amphibian-chytrid fungus system [25], [26], [27]. Ultimately, models predicting population declines and species extinctions must be parameterized with information about transmission, mortality, and recovery of susceptible individual in populations. Fundamental to understanding such complexity is a fine-scale and carefully standardized experimental approach to examining individual, species, and population level differences in host responses to infection.

## Supporting Information

Table S1 ***Batrachochytrium dendrobatidis*** **infection load comparisons (ANCOVA or Welch’s t-test) in larval amphibians by species, treatment, and sampling time-point.** Abbreviations are used for species: PR = *Pseudacris regilla*; RC = *Rana cascadae*; AB = *Anaxyrus boreas*. Abbreviations for treatments: H = high dose; I = intermediate dose; L = low dose; d = day. NS = non-significant comparisons; MS = marginally significant result (p<0.1). NA indicates that the comparison is not applicable because of low sample size (due to mortality or Bd-negative animals that could not be included in analyses). *Only 1 individual tested positive for infection in the low treatment group so the comparison of interest is high versus intermediate treatment on day 8 of the experiment made with a Welch’s t-test. **Only 1 individual in the low and the intermediate treatment tested positive for infection so no statistical comparisons among treatments were made. ***Rana cascadae was not included in species comparisons because only 1 individual tested positive for infection at this time-point/treatment combination.
(DOCX)

Table S2 ***Batrachochytrium dendrobatidis*** **infection load comparisons (ANCOVA or Welch’s t-test) in post-metamorphic amphibians by species, treatment, and sampling time-point.** Abbreviations are used for species: PR = *Pseudacris regilla*; RC = *Rana cascadae*; AB = *Anaxyrus boreas*. Abbreviations for treatments: H = high dose; I = intermediate dose; L = low dose. NS = non-significant comparisons; MS = marginally significant comparison (p<0.1). NA indicates that the comparison is not applicable because of low sample size (due to mortality or Bd-negative animals that could not be included in analyses). *Only 1PR remained alive by day 15 of the experiment and so was excluded from statistical analyses. **Only 1 AB remained alive by day 15 of the experiment and so was excluded from statistical analyses. ***No among species comparison on day 15 possible because only 1individual alive in PR and AB.
(DOCX)
