# Supplementary material for: Host Identity Matters in the Amphibian-Batrachochytrium dendrobatidis System: Fine-Scale Patterns of Variation in Responses to a Multi-Host Pathogen
Source: PLoS One. 2013 Jan 24;8(1):e54490. doi: 10.1371/journal.pone.0054490 (PMC3554766; doi:10.1371/journal.pone.0054490)
Supplement: Table S1 — Batrachochytrium dendrobatidis infection load comparisons (ANCOVA or Welch’s t-test) in larval amphibians by species, treatment, and sampling time-point. Abbreviations are used for species: PR = Pseudacris regilla; RC = Rana cascadae; AB = Anaxyrus boreas. Abbreviations for treatments: H = high dose; I = intermediate dose; L = low dose; d = day. NS = non-significant comparisons; MS = marginally significant result (p<0.1). NA indicates that the comparison is not applicable because of low sample size (due to mortality or Bd-negative animals that could not be included in analyses). *Only 1 individual tested positive for infection in the low treatment group so the comparison of interest is high versus intermediate treatment on day 8 of the experiment made with a Welch’s t-test. **Only 1 individual in the low and the intermediate treatment tested positive for infection so no statistical comparisons among treatments were made. ***Rana cascadae was not included in species comparisons because only 1 individual tested positive for infection at this time-point/treatment combination. (DOCX) [file pone.0054490.s001.docx]

**Supplementary Table 1**

| **Level** | **Overall comparison** | **Overall effect** | **Pair-wise comparison** | **Pairwise p-value** |
| --- | --- | --- | --- | --- |
| **Among treatment** | PR – d8 | ANCOVA F_3,18_-2.68;p=0.09 | NA | NS |
|  | PR – d15 | ANCOVA F_3,14_=5.93;p=0.01 | H vs L | <0.05 |
|  | RC – d8 | Welch’s t-test*(NS) | NS | NS |
|  | RC – d15 | NA** | NA | NA |
|  | AB – d8 | ANCOVAF_3,27_=6.47;p=0.005 | H vs L | <0.05 |
|  | AB – d15 | ANCOVAF_3,12_=5.18;p=0.023 | H vs I | <0.05 |
| **Between time-points** | PR - High | Welch’s t-test t_15_= -3.15;p=0.01 | NA | 0.01 |
|  | PR -Intermediate | Welch’s t-test (NS) | NA | NS |
|  | PR -Low | Welch’s t-test (NS) | NA | NS |
|  | RC - High | Welch’s t-test t5=-3.42;p=0.02 | d8 vs d15 | 0.02 |
|  | RC -Intermediate | Welch’s t-test (NS) | NS | NS |
|  | RC - Low | Welch’s t-test (NS) | NS | NS |
|  | AB – High | Welch’s t-test t_18_=-2.82;p=0.01 | d8 vs d15 | 0.01 |
|  | AB –Intermediate | Welch’s t-test (NS) | NS | NS |
|  | AB – Low | Welch’s t-test (NS) | NS | NS |
| **Among species** | Day 8 High | ANCOVAF_3,22_=12.7;p=0.0002 | PR vs RC | NS |
|  |  |  | PR vs AB | <0.05 |
|  |  |  | AB vs RC | <0.05 |
|  | Day 15 High | ANCOVA (NS) | All NS | NS |
|  | Day 8 Intermediate | ANCOVA (NS) | All NS | NS |
|  | Day 15 Intermediate | Welch’s t-test*** | NS | NS |
|  | Day 8 Low | Welch’s t-test*** | NS | NS |
|  | Day 15 Low | Welch’s t-test*** | NS | NS |
