# Supplementary material for: Host Identity Matters in the Amphibian-Batrachochytrium dendrobatidis System: Fine-Scale Patterns of Variation in Responses to a Multi-Host Pathogen
Source: PLoS One. 2013 Jan 24;8(1):e54490. doi: 10.1371/journal.pone.0054490 (PMC3554766; doi:10.1371/journal.pone.0054490)
Supplement: Table S2 — Batrachochytrium dendrobatidis infection load comparisons (ANCOVA or Welch’s t-test) in post-metamorphic amphibians by species, treatment, and sampling time-point. Abbreviations are used for species: PR = Pseudacris regilla; RC = Rana cascadae; AB = Anaxyrus boreas. Abbreviations for treatments: H = high dose; I = intermediate dose; L = low dose. NS = non-significant comparisons; MS = marginally significant comparison (p<0.1). NA indicates that the comparison is not applicable because of low sample size (due to mortality or Bd-negative animals that could not be included in analyses). *Only 1PR remained alive by day 15 of the experiment and so was excluded from statistical analyses. **Only 1 AB remained alive by day 15 of the experiment and so was excluded from statistical analyses. ***No among species comparison on day 15 possible because only 1individual alive in PR and AB. (DOCX) [file pone.0054490.s002.docx]

**Supplementary Table 2**

| **Level** | **Overall comparison** | **Overall effect** | **Pair-wise comparison** | **Pairwise p-value** |
| --- | --- | --- | --- | --- |
| **Among treatment** | PR – d8 | ANCOVAF_3,19_=14.4;p=0.0001 | H vs L | <0.05 |
|  |  |  | I vs L | <0.05 |
|  |  |  | H vs I | NS |
|  | PR – d15 | Welch’s t-test*; t_6_=4.314;p=0.03 | I vs L | 0.03 |
|  | RC – d8 | ANCOVA (NS) | NS | NS |
|  | RC – d15 | ANCOVA (NS) | NS | NS |
|  | AB – d8 | ANCOVA F3,17=14.5;p=0.0002 | H vs L | <0.05 |
|  |  |  | I vs L | <0.05 |
|  |  |  | H vs I | NS |
|  | AB – d15 | Welch’s t-test (NS) | NA | NS |
| **Between time-points** | PR - High | NA* | NA | NA |
|  | PR - Intermediate | Welch’s t-test;t_8_=3.57;p=0.004 | d8 vs d15 | 0.004 |
|  | PR - Low | Welch’s t-test;t_12_=2.22;p=0.09 | d8 vs d15 | MS |
|  | RC - High | Welch’s t-test (NS) | NA | NS |
|  | RC - Intermediate | Welch’s t-test (NS) | NA | NS |
|  | RC - Low | Welch’s t-test t_5_=3.568;p=0.020 | d8 vs d15 | 0.020 |
|  | AB – High | NA** | NA | NA |
|  | AB – Intermediate | Welch’s t-test t_11_=-2.48;p=0.040 | NA | 0.040 |
|  | AB – Low | Welch’s t-test t_10_=-4.87;p<0.0007 | NA | 0.0007 |
| **Among species** | Day 8 High | ANCOVA F_3,18_=61.1;p<0.0001 | PR vs RC | <0.05 |
|  |  |  | PR vs AB | <0.05 |
|  |  |  | AB vs RC | <0.05 |
|  | Day 15 High | NA*** | NA | NA |
|  | Day 8 Intermediate | ANCOVA F_3,18_=58.5;p<0.0001 | PR vs RC | <0.05 |
|  |  |  | PR vs AB | <0.05 |
|  |  |  | AB vs RC | <0.05 |
|  | Day 15 Intermediate | ANCOVA F_3,6_=28.3;p<0.0008 | PR vs RC | <0.05 |
|  |  |  | PR vs AB | NS |
|  |  |  | AB vs RC | <0.05 |
|  | Day 8 Low | ANCOVA F_3,17_=17.2;p<0.0001 | PR vs RC | <0.05 |
|  |  |  | PR vs AB | <0.05 |
|  |  |  | AB vs RC | <0.05 |
|  | Day 15 Low | ANCOVA F_3,9_=59.3;p<0.0001 | PR vs RC | NS |
|  |  |  | PR vs AB | <0.05 |
|  |  |  | AB vs RC | <0.05 |
